# A chicken model of pharmacologically-induced Hirschsprung disease reveals an unexpected role of glucocorticoids in enteric aganglionosis

**DOI:** 10.1242/bio.201410454

**Published:** 2015-04-02

**Authors:** Jean-Marie Gasc, Maud Clemessy, Pierre Corvol, Hervé Kempf

**Affiliations:** 1 Centre Interdisciplinaire de Recherche Biomédicale (CIRB), Collège de France, 75005 Paris, France; 2 Chaire de Médecine Expérimentale, Collège de France, 75005 Paris, France; 3 Centre de Recherche St-Antoine UMRS-938, INSERM-Université Pierre et Marie Curie, Paris 6, 75012 Paris, France; 4 UMR 7365 CNRS-Université de Lorraine, IMoPA, Faculté de Médecine, 54500 Vandoeuvre-lès-Nancy, France

**Keywords:** Hirschsprung disease, Endothelin, Chick embryo, Enteric nervous system, Glucocorticoids

## Abstract

The enteric nervous system originates from neural crest cells that migrate in chains as they colonize the embryonic gut, eventually forming the myenteric and submucosal plexus. Failure of the neural crest cells to colonize the gut leads to aganglionosis in the terminal gut, a pathological condition called Hirschsprung disease (HSCR) in humans, also known as congenital megacolon or intestinal aganglionosis. One of the characteristics of the human HSCR is its variable penetrance, which may be attributable to the interaction between genetic factors, such as the endothelin-3/endothelin receptor B pathway, and non-genetic modulators, although the role of the latter has not well been established. We have created a novel HSCR model in the chick embryo allowing to test the ability of non-genetic modifiers to alter the HSCR phenotype. Chick embryos treated by phosphoramidon, which blocks the generation of endothelin-3, failed to develop enteric ganglia in the very distal bowel, characteristic of an HSCR-like phenotype. Administration of dexamethasone influenced the phenotype, suggesting that glucocorticoids may be environmental modulators of the penetrance of the aganglionosis in HSCR disease.

## INTRODUCTION

The enteric nervous system (ENS) is formed from neural crest cells (NCCs) (Burns, 2005; Goldstein et al., 2013). Vagal NCCs first migrate dorso-laterally from the neural tube, then enter the foregut and finally migrate along the length of the digestive tube in a rostro-caudal direction (Burns and Le Douarin, 2001; Burns, 2005; Goldstein et al., 2013). In addition, sacral NCCs colonize the very distal portion of the gut through an opposite caudo-rostral migration, thereby contributing to a small portion of the terminal ENS (Burns and Douarin, 1998; Burns, 2005; Erickson et al., 2012; Goldstein et al., 2013). Concomitantly to their migration, these NCCs proliferate and differentiate into neurons and glial cells to eventually form up to the very end of the gut a double ganglionic chain consisting of the outer myenteric plexus and the inner submucosal plexus. Enteric NCC migration, proliferation, differentiation and/or survival are regulated by proteins and receptors that are expressed by the NCCs or the gut mesenchyme (Burns, 2005; Goldstein et al., 2013). Mutations in genes encoding for these factors impede the formation of functional enteric ganglia and result in aganglionosis, a condition called Hirschsprung disease (HSCR) in humans, where the absence of the terminal ENS leads to a deficiency of peristaltic movements over various segments of the hindgut and results in bowel obstruction (Amiel et al., 2008; Goldstein et al., 2013). With an incidence of 1 in 5000 live births, it is a rare but life-threatening pathology if untreated. The surgical ablation at birth of the aganglionic and atonic segment is yet the only remedy to prevent the obstruction of the intestine leading to the formation of a megacolon. HSCR is a complex, polygenic congenital disorder with non-Mendelian inheritance and male predominance. Interestingly, it is also characterized by incomplete penetrance and variable severity (Amiel et al., 2008). This is partly explained by the combination of mutations in different key genes that are individually responsible for HSCR and the role of additional modifier genes, which all together contribute to an intricate but coordinated network regulating enteric NCC development (Amiel et al., 2008; Wallace and Anderson, 2011; Goldstein et al., 2013).

The members of the endothelin family are crucial regulators of NCC development. This family is composed of three peptides called endothelin-1 (EDN1), endothelin-2 (EDN2) and endothelin-3 (EDN3) encoded by three distinct genes (Inoue et al., 1989). They are synthesized as pro-hormones (big-EDNs) and processed into active endothelins by the endothelin-converting enzyme (ECE1). The endothelin effects are mediated through two G-coupled protein receptors in mammals, endothelin receptor A (EDNRA) and endothelin receptor B (EDNRB), with distinct and specific binding affinities for native and synthetic ligands (Sakurai et al., 1992). EDNRA has a high affinity for EDN1 and EDN2 and a low affinity for EDN3 (Arai et al., 1990), whereas EDNRB does not discriminate between the three endothelin isoforms (Sakurai et al., 1990). Rodents with natural or targeted mutations for *Edn3* (Baynash et al., 1994), *Ednrb* (Hosoda et al., 1994; Gariepy et al., 1996) or *Ece1* (Yanagisawa et al., 1998) exhibit severe aganglionosis in the distal colon, similar to that observed in humans where mutations in genes encoding for members of the endothelin family account for approximately 5% of HSCR cases (Amiel et al., 2008). Interactions between EDNRB and Sox10 have been shown to modulate the penetrance and severity of aganglionosis (Cantrell et al., 2004). The genetic background can also impact on these features in an *Ednrb*-deficient HSCR model (Dang et al., 2011), suggesting that modifiers genes could potentially alter the phenotypic expression of HSCR such as first shown with *Lcam1* for the endothelin family member (Wallace et al., 2011). Finally, non-genetic factors may also play a role in the variable expression of HSCR, but have been hardly explored (Fu et al., 2010) because the specific contribution of such modifiers in congenital malformation is challenging to study in humans and even in mouse models.

In order to provide a straightforward system to test non-genetic factors that would potentially modify the penetrance of aganglionosis, we sought to develop a model where an HSCR-like phenotype could be easily and quickly induced. For this purpose, we chose the chick embryo, a model free of maternal influence, in which we pharmacologically disrupted the establishment of a functional ENS through administration of phosphoramidon, an inhibitor of ECE1. Using this novel instrumental model of HSCR, we found a gender effect in the expression of the induced-disease, similar to the sex imbalance observed in human HSCR, and that the synthetic glucocorticoid dexamethasone inversely altered the HSCR phenotype according to the sex of the chick embryos.

## MATERIALS AND METHODS

### Embryos, drug administration and autopsy

Fertilized eggs of the White Leghorn chicken strain (Haas, Kalten House, France) were incubated at 38°C under high humidity conditions. Embryos were staged by the number of hours or days following incubation. At the time specified for each experimental group, we performed shell-less culture of the control and treated chicken embryos according to the original protocol (Auerbach et al., 1974). This culture technique not only allowed the embryos to be readily treated with the drug(s) of interest but also to interrupt the treatment at any time by blotting the oil suspension with a small piece of sterile filter paper.

All endothelin receptor antagonists used in this study were generous gifts obtained either from Hoffman-La Roche (Ro antagonists) or Hoechts Marion Roussel (RU antagonists) and characterized by the respective company as ETA-specific (RU69986), ETB-specific (RU70337) and dual ETA/ETB (Ro48-5695, Bosentan) in Mammals. Endothelin receptor antagonists, ECE1 (phosphoramidon) and NEP (thiorphan) inhibitors (Sigma), EDN1, EDN3 (Bachem) and dexamethasone (Sigma) were administered as a 25 µl suspension in sterile mineral oil as previously described (Kempf et al., 1998).

The Petri dish containing the treated embryo was returned to the incubator until day 10 (E10), a stage when, during normal development, the NCC-derived neurons have entirely colonized up to the most distal segment of the gut and when gross anatomical observation for possible malformation of craniofacial skeleton may be used to evaluate the results of the endothelin system inactivation (Kempf et al., 1998).

The procedures for the care and killing of the animals were in accordance with the European Community regulations.

### Immunohistochemistry and RNA *in situ* hybridization

The embryos were fixed overnight in 4% paraformaldehyde. After dehydration in graded series of ethanol and butanol, embryos were embedded in paraffin and sagittal 7-µm sections were mounted on silanized slides for further histological analysis.

Neurons of neural crest origin in the gut were characterized by immunolocalization with the anti-HNK1 mouse monoclonal antibody (1/3000, C0678, Sigma, France) following a routine protocol using an ABC Elite Avidin-Biotin-Peroxidase kit (Vector Laboratories, Burlingame, California). *In situ* hybridization was performed as previously described (Sibony et al., 1995) using ^35^S-UTP-labeled antisense riboprobe against chick *Ednrb* (Kempf et al., 1998). Sections were examined and photographed using a Leica microscope equipped with a Leica DFC420 camera.

### Inclusion criteria and statistical analysis

Each egg was given a number, which identified it to its treatment group. At the end of the experiment, the anatomical and histological observations of the embryos were made blindly without knowledge of the treatment received by the embryos. Only embryos alive at the time of observation were included.

Data are represented in contingency table indicating the percentage of embryos presenting malformations. Corresponding number of malformed embryos per total number of embryos are also shown in parentheses. Statistical differences were assessed by χ^2^ analysis.

## RESULTS

### Pharmacological inhibition of ECE1 induces aganglionosis

**Fig. 1.**
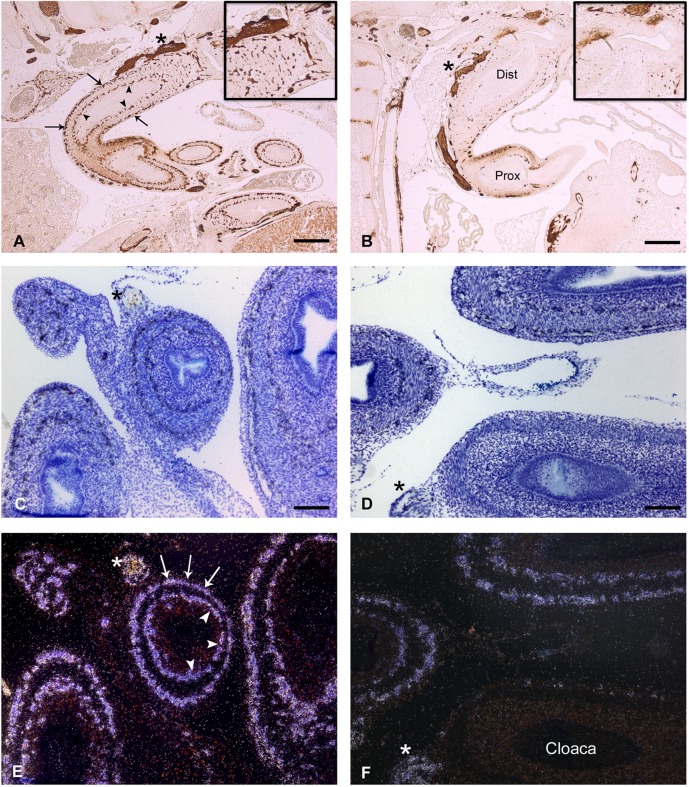
Distribution of NCC derived ganglionic cells in the terminal segment of the gut of chick embryo at E10. Immunostaining with NCC specific HNK1 antibody (A,B) and *in situ* hybridization with c*Ednrb* ^35^S-riboprobe (C–F). (A,C,E) Both immunostaining and *in situ* labeling reveal the same cell distribution of enteric neurons in a control embryo in organized neuronal plexuses (Meissner plexus, outer circle, arrows; Auerbach plexus, inner circle, arrowheads). (B,D,F). Phosphoramidon-treated embryo display normal pre-coecal distribution of ganglionic cells, but absence of terminal ganglionic cells in cloacal portion of the treated embryos, characteristic of HSCR syndrome. (A–D) Bright-field illumination; (E,F) dark-field illumination. Dist: distal part of the hindgut; Prox: proximal part of the hindgut. Small inserts in both A and B panels are magnifications of the most distal portion of the gut. Asterisks are apposed next to the nerve of Remak. Bar: 150 µm (A,B); 75 µm (C,D).

In E10 control chick embryos, the NCC-derived enteric ganglia are formed in the embryonic distal gut. Between the caecum and the cloaca, the HNK1 antibody labeled all NCCs within the myenteric and submucosal plexuses as well as the avian-specific nerve of Remak on the dorsal side of the cloaca, derived from sacral NCCs (Fig. 1A) (Doyle et al., 2004; Nagy et al., 2012). Both plexuses also strongly expressed *Ednrb* mRNA (Fig. 1C,E) (Nataf et al., 1996; Nagy and Goldstein, 2006).

Our strategy of pharmacological inhibition of ECE1 by phosphoramidon was expected to reduce the generation of mature endothelin peptides in the chick embryo, and consequently silence both EDNRA and EDNRB, which are required to build a normal craniofacial skeleton and establish a complete enteric nervous system, respectively. The effects of phosphoramidon administration were thus evaluated by the percentage of E10 embryos displaying typical malformations resulting from an inactivated endothelin system, i.e. a craniofacial phenotype characterized anatomically by a reduced or absent lower beak (see Kempf et al., 1998 for details), and an intestinal or HSCR phenotype that refers to the absence of sympathetic ganglia. Compared to controls, the first and apparent phenotype of phosphoramidon-treated chicken embryos was an atrophied lower beak (supplementary material Fig. S1). Further histological examination showed that signals for both HNK1 immunostaining (Fig. 1B) and *Ednrb* mRNA *in situ* hybridization (Fig. 1D,F) were almost completely abolished in the cloaca of the treated embryos, with a total absence of enteric neurons in the submucosal plexus and few remaining cells in the myenteric one. Colorectal portion of the hindgut was less affected with a caudo-rostral penetrance. Interestingly, the sacral-derived nerve of Remak was still strongly HNK1-positive. These observations clearly established that phosphoramidon administration impaired vagal NCC development that eventually led to an absence of organized enteric plexuses beyond the caecum, typical of HSCR. The percentage of embryos that displayed this phenotype increased with the inhibitor concentration as treatment with phosphoramidon at 0.33, 0.50 or 1 mg/ml generated 0%, 37% or 46% of aganglionic embryos, respectively (Table 1; data not shown).

**Table 1.**
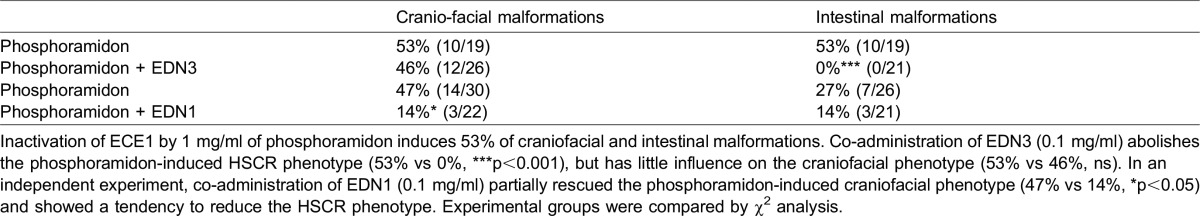
Functional rescue of phosphoramidon treated embryos by EDN3 and EDN1

### Phosphoramidon-induced HSCR phenotype is due to the specific blockade of the EDN3/EDNRB pathway

As phosphoramidon is not a pure ECE1 inhibitor, the induced malformations, though characteristic of endothelin deprivation, could be the consequence of the inhibition of another related metallo-enzyme, such as neprilysin also denominated neutral endopeptidase (NEP). However, administration of thiorphan, a potent inhibitor of NEP but not of ECE1, did not induce any craniofacial or intestinal phenotype, even at very high concentration (5 mg/ml; supplementary material Table S1). Most strikingly, when embryos treated by a dose of phosphoramidon were supplemented with EDN3, the percentage of embryos with HSCR phenotype dropped from 53% to 0%. Noteworthy, there was no significant modification in the number of embryos with craniofacial malformations, indicating that the addition of EDN3 fully and specifically restored a normal intestinal phenotype, but has hardly any effect on the craniofacial phenotypes (Table 1). In contrast, both HSCR and craniofacial phenotypes were partially restored when phosphoramidon-treated embryos were supplemented with exogenous EDN1 instead of EDN3 (Table 1).

### Time-dependence of NCC neurons to endothelins

Previous studies suggested there is a temporally limited and narrow period of sensitivity of NCCs to the endothelin signaling pathways, both in chick embryos (Kempf et al., 1998) and in the mouse fetuses (Shin et al., 1999). In order to determine the most sensitive stage of ET signaling that induced intestinal phenotype in the present model, we took advantage of the shell-less culture to administer phosphoramidon to the embryo at different development stages and terminate the treatment by washing out the medium (Table 2).

**Table 2.**
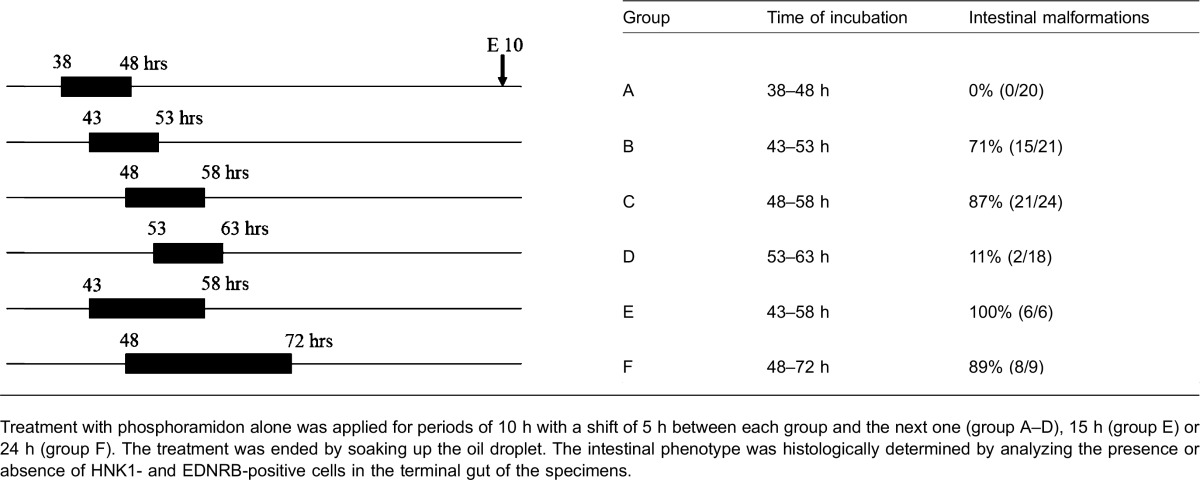
Characterization of the time window of EDN sensitivity

Embryos treated from 38 to 48 h (group A) and 53 to 63 h (group D) displayed no or very few individuals with HSCR phenotype. Groups B (43 to 53 h) and C (48 to 58 h) accounted for 71% and 87% HSCR phenotype malformations, respectively. 100% malformed embryos were observed at E10 in the period encompassing 43 to 58 h (group E). Finally, a group of embryos that was kept untreated up to 48 h and then treated for 24 h gave identical results to that obtained for the Group C (87% and 89%). Altogether theses results showed that the time period of sensitivity to endothelins of NCC-derived neurons is a very early and brief event as the maximum effect happened between 43 and 58 h after incubation.

### Sex imbalance of HSCR phenotype

In all the results reported above, the embryos of both sexes were treated without knowing a priori their gender, since in this species the sexual differentiation of the gonads occurs around E7.5, i.e. days after the end of the period of sensitivity of the NCC-derived ganglionic cells to endothelins. However, it is possible to individually determine the sex of the embryos either at early stages by molecular means or late stages by morphological examination of their gonads (Clinton et al., 2001; Chue and Smith, 2011). As our specimens were all autopsied at E10, we thus a posteriori classified phosphoramidon-induced terminal gut malformations according to gender using morphological discrimination. This further investigation established that, if the rate of malformations of the colon was 52% for the entire group (males and females), the penetrance rate was 65% for males and 38% for females (Table 3), demonstrating a sex difference in our model.

**Table 3.**
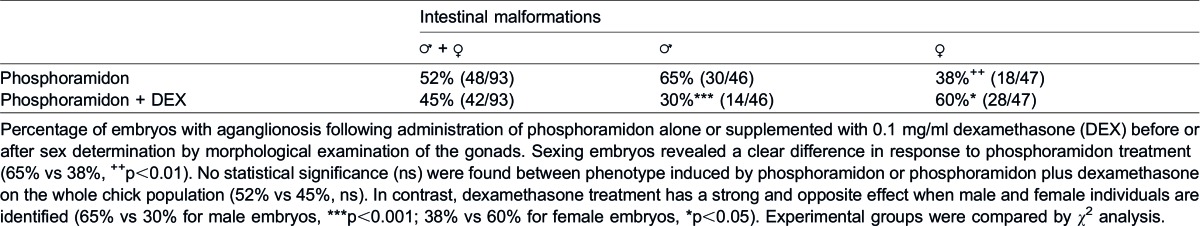
Sex-dependency and influence of glucocorticoids

### Dexamethasone: a modifier factor of the EDN3/EDNRB pathway

Glucocorticoids have been reported to alter proliferation, migration and differentiation of NCC-derived neurons (Smith and Fauquet, 1984; Ross et al., 1995). On the other hand, glucocorticoids are known to be subjected to pathophysiological variations and their levels can reach acute peaks during gestational stress periods for instance (Cottrell and Seckl, 2009). Altogether, this prompted us to explore whether a glucocorticoid molecule such as dexamethasone could interfere with the penetrance of endothelin-specific HSCR type malformations in our chick model. No craniofacial or intestinal malformations were observed with increasing concentrations of dexamethasone alone (data not shown). When dexamethasone was applied in phosphoramidon-treated embryos, we noticed a slight decrease in the percentage of malformed embryos (Table 3). However, when re-analyzed according to the sex of the embryos as described above, the data surprisingly revealed that dexamethasone treatment has striking and opposite effects in male and in female embryos: the male penetrance after treatment with phosphoramidon alone was significantly inverted into a female penetrance in the group treated with phosphoramidon and dexamethasone (Table 3).

## DISCUSSION

The chick embryo model is a simple and useful alternative to complex, expensive and time-consuming transgenic studies in mammals. For that reason, it has been extensively used in developmental studies, particularly those dealing with NCC development. The main objective of our study was to create a simple, rapid and reproducible HSCR model in the chick in order to explore potential factors, which may modify the phenotype. As a disrupted endothelin system has been shown to be responsible for the development of HSCR in humans and in mice (Baynash et al., 1994; Hosoda et al., 1994; Gariepy et al., 1996; Yanagisawa et al., 1998), in order to knock-down the EDN3/EDNRB pathway in the chick embryo, we built on a strategy similar to the one we have previously and successfully used for the EDN1/EDNRA pathway (Kempf et al., 1998). In the present study, we used shell-less culture (Auerbach et al., 1974), a technique that allowed us to be more precise to deposit the oil-made drug reservoir onto the embryo and also interrupt the treatment whenever required.

The lack of a full-length chick *Ednrb* cDNA at the time of this study precluded direct pharmacological studies aimed at selecting a specific EDNRB antagonist. To circumvent this difficulty, we tested several compounds known to bind and antagonize the EDNRB receptor, either by tissue binding/displacement experiments (data not shown) or by exploring *in ovo* their ability to inhibit ENS formation (supplementary material Table S1). None of the mammalian antagonists screened displayed any EDNRB antagonist properties in the chick, a situation already encountered for other chick receptors towards mammalian antagonists (Kempf et al., 1996; Schroeder et al., 1997). We then reasoned that an alternative to block the EDNRB pathway was to deprive the embryo of the endogenous agonist of this receptor, i.e. EDN3. We chose to inhibit ECE1, which processes inactive big-endothelins into active endothelins, in order to reproduce the effects of a treatment with a mixed endothelin receptor antagonist or those observed after *Ece1* genomic invalidation (Yanagisawa et al., 1998). This experimental strategy led to an absence of HNK1/*Endrb* positive enteric ganglia, with the exception of sporadic cells in the outer plexus of the very distal bowel, which were apparently unaffected by the treatment but unable to form fully organized and functional enteric neurons. Those few cells are most probably of sacral origin as the sacral NCC-derived nerve of Remak is also unaltered by the pharmacological inactivation of ECE1. Indeed, our findings are highly reminiscent of results obtained after surgical ablation of vagal NCCs where the terminal hindgut was shown to be free from enteric plexus, even though isolated neural cells from sacral origin were found to occur but failed to compensate for the lack of enteric plexus (Burns et al., 2000; Donnell et al., 2005). A more detailed exploration of the impact of our different treatments may be necessary to draw definitive conclusion, especially regarding the length of the segments affected through the expression analysis of additional enteric markers such as the transcriptional factor Sox10, known to be regulated by (Zhu et al., 2004) and interact (Cantrell et al., 2004; Stanchina et al., 2006) with END3/ENDRB signaling during enteric development. However, in comparison to surgical ablation procedures that also made use of HNK1 staining to characterize resulting aganglionosis (Burns et al., 2000; O'Donnell and Puri, 2009), our approach of ECE1 pharmacological inhibition by phosphoramidon proved to be an efficient and straightforward procedure in the chick to induce a reproducible and dose-dependent loss of enteric ganglia in the very distal portion of the chicken gut, an intestinal phenotype that recapitulates that of human HSCR resulting from *EDN1* or *ENDRB* mutations.

Because our strategy also depleted the embryo of all mature endothelins, a craniofacial phenotype (reduction or absence of lower beak) similar to the one obtained after genetic or pharmacological inactivation of the EDN1/ENDRA was also observed (Hosoda et al., 1994; Kurihara et al., 1994; Gariepy et al., 1996; Kempf et al., 1998; Yanagisawa et al., 1998; Clouthier et al., 2000). This allowed us to anticipate the treatment efficacy on intestinal phenotype by a straightforward anatomical observation of the head of the embryos at E10. But more importantly, this strongly suggested that the craniofacial and intestinal phenotypes obtained after phosphoramidon administration were typical of EDNRA and EDNRB inactivation, respectively (Hosoda et al., 1994; Kurihara et al., 1994; Gariepy et al., 1996; Kempf et al., 1998; Yanagisawa et al., 1998; Clouthier et al., 2000). However, phosphoramidon is not a specific ECE1 inhibitor, as it can also block other Zinc-metallopeptidases such as NEP. Therefore, we designed a series of experiments to show that the phosphoramidon-induced HSCR phenotype was specific and not due to alternate or combinatory inhibition of another enzyme. First, the response to phosphoramidon was dose-dependent substantiating against a stochastic response. Second, supplementation with exogenous END3 totally rescued the intestinal phenotype, showing that the HSCR malformations observed after ECE1 inhibition by phosphoramidon were due to the specific deprivation of the natural endogenous peptide. Third, thiorphan administration did not induce any phenotype, ruling out an effect of NEP. Fourth, gross anatomical observation did not reveal any obvious malformation in organs other than craniofacial skeleton and intestine. Altogether, these data validate our strategy of hormonal deprivation through pharmacological inhibition of ECE1 using phosphoramidon, as an easy and reproducible model of HSCR-like phenotype in the chick. Interestingly, a marked sex difference in the phenotype expression was revealed in our chick model, in contrast to mouse models that do not display a sex bias, except in complex oligogenic models (McCallion et al., 2003).

The variability of the penetrance and severity of HSCR still remains an open question. Although numerous reports have shown that combination of gene mutations and participation of modifier genes contribute to this variability, non-genetic factors may be also involved. Indeed, it has been largely documented that undesirable metabolic, hormonal or environmental factors occurring during fetal life may contribute to the occurrence of diseases later in life. However, although such ‘developmental programming’ is now widely recognized (Gluckman et al., 2008), it has not been explored for HSCR with the exception of a recent experimental report on vitamin A (Fu et al., 2010). Interestingly using our model, we provide evidence that the penetrance of enteric defects in phosphoramidon-treated chick embryos is sensitive to glucocorticoid treatment in a sex-dependent manner, as dexamethasone, a pure glucocorticoid molecule in avian species (Groyer et al., 1985), was able to inverse the sex-related penetrance seen after phosphoramidon-induced HSCR. Although we did not address how glucocorticoid and endothelin signalings can interact with each other in neural crest-derived neurons, it might be of interest to further investigate this issue as it has been reported that glucocorticoids can promote *Ednrb* up-regulation in epithelial cells (Zhang et al., 2003). Our results support the hypothesis that abnormal levels of glucocorticoids could be an environmental modifier in human HSCR diseases. This adds up to the growing number of reports showing that glucocorticoids play a role in early developmental programming in cardiovascular and neurological disorders (O'Regan et al., 2001; O'Regan et al., 2004; O'Regan et al., 2008; Khulan and Drake, 2012), where sex differences also start to be described (Intapad et al., 2014).

In conclusion, using a new model of HSCR in the chick, our data suggest for the first time that stress-induced glucocorticoids during pregnancy, a pathophysiological condition frequently encountered but whose impact is variable and consequences not too deleterious to keep the embryo alive until birth (Cottrell and Seckl, 2009), could be associated with different penetrance in infants genetically predisposed to aganglionosis.

Our primary and unique goal of the current work was to report an original and simple pharmacologically-induced model of Hirschsprung disease in the chick that, compared to previous models of surgical ablation for instance, is very straightforward to replicate. We believe that, as detailed here for glucocorticoids, the model might be used to easily test additional factors suspected to potentially affect the severity and/or penetrance of the HSCR disease. Additionally, although beyond the scope of the descriptive report herein, this model offers the opportunity to investigate the impact of END3/ENDRB signaling blockade on enteric development, including proliferation, differentiation or survival. As such, it may provide an original and complementary approach to more detailed studies habitually performed to address these questions such as deficient-mice or organ culture assays (Nagy and Goldstein, 2006).

## Supplementary Material

Supplementary Material
