# Supplementary material for: A chicken model of pharmacologically-induced Hirschsprung disease reveals an unexpected role of glucocorticoids in enteric aganglionosis
Source: Biol Open. 2015 Apr 2;4(5):666–71. doi: 10.1242/bio.201410454 (PMC4434818; doi:10.1242/bio.201410454)
Supplement: Supplementary Material [file supp_4_5_666__index.html]

A chicken model of pharmacologically-induced Hirschsprung disease reveals an unexpected role of glucocorticoids in enteric aganglionosis — A chicken model of pharmacologically-induced Hirschsprung disease reveals an unexpected role of glucocorticoids in enteric aganglionosis — Supplementary Material 

# A chicken model of pharmacologically-induced Hirschsprung disease reveals an unexpected role of glucocorticoids in enteric aganglionosis

## bio.201410454 Supplementary Material

**Files in this Data Supplement:**

- Supplementary Material - Jean-Marie Gasc et al. doi: 10.1242/bio.201410454
