# Supplementary material for: A chicken model of pharmacologically-induced Hirschsprung disease reveals an unexpected role of glucocorticoids in enteric aganglionosis
Source: Biol Open. 2015 Apr 2;4(5):666–71. doi: 10.1242/bio.201410454 (PMC4434818; doi:10.1242/bio.201410454)
Supplement: Supplementary Material [file supp_bio.201410454_bio.201410454-s1.pdf]

Supplementary Material
Jean-Marie Gasc et al. doi: 10.1242/bio.201410454

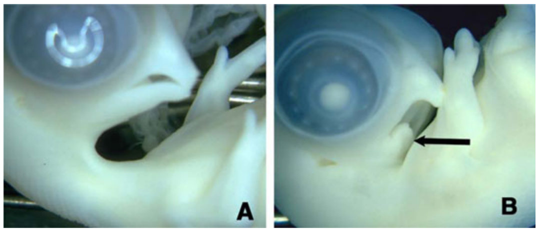

Fig. S1. Craniofacial malformations following phosphoramidon treatment. External appearance of the head of control (A) and 1 mg/ml phosphoramidon-treated (B) embryos. Reduced lower beak formation induced by pharmacological inhibition of the endothelin system is marked by an arrow in panel B.

Table S1. Phenotypes obtained with various antagonists or inhibitors of the endothelin system

		Cranio-facial malformations	Intestinal malformations
Vehicle	Oil	0% (0/10)	0% (0/10)
Antagonists			
ETA	RU69986 (2 mg/ml)	80% (8/10)	0% (0/8)
ETB	RU70337 (0.3 mg/ml)	75% (3/4)	0% (0/3)
Mixed	Ro48-5695 (0.3 mg/ml)	100% (4/4)	0% (0/3)
Mixed	Bosentan (3 mg/ml)	86% (6/7)	0% (0/7)
Inhibitors	Phosphoramidon (1 mg/ml)	55% (6/11)	46% (6/13)
	Thiorphan (5 mg/ml)	0% (0/7)	0% (0/5)

Binding specificity as determined by bioactivity *in ovo* in the chick embryo under the experimental conditions as described in the Materials and Methods section. None of the mammalian antagonists reagents tested was effective in inducing intestinal malformations in the chicken, contrary to phosphoramidon, an inhibitor of ECE-1, which inhibited half the biological activity at 1 mg/ml.

Biology Open
